# Monitoring the effect of antiviral treatment on the development of cirrhosis and hepatocellular carcinoma in chronic hepatitis B patients

**DOI:** 10.1590/1806-9282.20240803

**Published:** 2024-12-02

**Authors:** Kamil Mert, Bengu Tatar, Yasemin Nadir, Suheyla Serin Senger

**Affiliations:** 1 Malazgirt State Hospital, Department of Infectious Diseases and Clinical Microbiology – Muş, Turkey.; 2 University of Health Sciences, Izmir Faculty of Medicine, Department of Infectious Diseases and Clinical Microbiology – İzmir, Turkey.; 3 University of Health Sciences, Izmir Tepecik Education and Research Hospital, Department of Infectious Diseases and Clinical Microbiology – İzmir, Turkey.

**Keywords:** Hepatocellular carcinoma, Liver fibrosis, Cirrhosis, Antiviral drugs, Chronic hepatitis B

## Abstract

**OBJECTIVE:**

Despite the availability of treatment and vaccines for chronic hepatitis B infection, it remains a public health problem. The use of nucleos(t)ide antiviral agents is effective in preventing complications such as cirrhosis and hepatocellular carcinoma, but despite the reduction, the risk persists. The objective of this study was to assess alterations in aspartate aminotransferase to platelet ratio index and Fibrosis-4 scores and examine the effectiveness of risk estimates for HCC in chronic hepatitis B scores in predicting the risk of hepatocellular carcinoma among chronic hepatitis B patients undergoing prolonged antiviral treatment.

**METHODS:**

This is a retrospective 10-year follow-up study that included chronic hepatitis B patients who received antiviral treatment. The alterations in aspartate aminotransferase to platelet ratio index and Fibrosis-4 scores were examined for the first, fifth, and tenth years. In addition, the expected long-term risk of hepatocellular carcinoma, if antiviral treatment was not initiated, was calculated using the risk estimates for HCC in chronic hepatitis B scores before treatment, and the incidence of hepatocellular carcinoma despite long-term antiviral treatment was compared with these values.

**RESULTS:**

The study included a total of 218 chronic hepatitis B patients. A noteworthy decline in aspartate aminotransferase to platelet ratio index and Fibrosis-4 scores was noted across all fibrosis groups, irrespective of the initial degree of fibrosis, during the first year of antiviral treatment. Following the calculation using risk estimates for HCC in chronic hepatitis B scores, one case was observed compared to the expected 15.04 cases of hepatocellular carcinoma.

**CONCLUSION:**

We showed that both aspartate aminotransferase to platelet ratio index and Fibrosis-4 scores significantly decreased until the fifth year of antiviral treatment, with a prominent decline occurring in the first year. This study demonstrates that the impact of antiviral treatment on fibrosis can be monitored using noninvasive fibrosis scores.

## INTRODUCTION

Despite the availability of effective treatment and vaccines for chronic hepatitis B (CHB) infection, it remains a significant public health problem on a global scale, including in our country. The unique genomic structure of the hepatitis B virus (HBV) enables its integration into the hepatocyte genome, leading to the inhibition of complete elimination of the virus^1^. Complications such as liver failure, cirrhosis, and hepatocellular carcinoma (HCC) may arise in those infected with HBV^2^. According to the guideline, treatment is indicated in cases of high viral load, elevated aminotransferase, and/or at least moderate necroinflammation^3^. The use of nucleos(t)ide antiviral agents is effective in preventing complications such as cirrhosis and HCC, but despite the reduction, the risk persists^4^.

While liver biopsy remains the gold standard for assessing the fibrosis level, noninvasive alternatives are preferred due to the absence of complications. Aspartate aminotransferase (AST) to platelet ratio index (APRI) and Fibrosis-4 (FIB-4) index are commonly used^5^. Additionally, specific scoring methods have been developed to predict the risk of HCC development. Notably, the risk estimates for HCC in chronic hepatitis B (REACH-B) scoring method have been designed to estimate the risk of HCC in treatment-naive CHB patients over 5 and 10 years^6^.

The objective of this study was to assess alterations in APRI and FIB-4 scores and examine the effectiveness of REACH-B scores in predicting the risk of HCC for patients receiving antiviral treatment.

## METHODS

This retrospective study was conducted on patients diagnosed with CHB who initiated treatment between January 2008 and January 2018. Inclusion criteria were: patients who received potent antiviral treatment (AVT) for a minimum of 5 years, who had histopathological examination results before treatment, <18 years old, those with a fibrosis score of ≥5 at baseline, individuals already diagnosed with HCC, and those with co-infections such as hepatitis C virus, human immunodeficiency virus, or hepatitis D virus were excluded. Stratified randomization was used in patient selection.

In the assessment of biochemical parameters, AST (U/L), alanin aminotransferase (ALT) (U/L), total bilirubin (mg/dL), direct bilirubin (mg/dL), and albumin (g/dL) were quantified using the AU5800 auto-analyzer (Beckman Coulter Inc., CA, USA). Alpha-fetoprotein (AFP) (ng/mL) levels were determined through the DxI 800 auto-analyzer (Beckman Coulter Inc., CA, USA). Prothrombin time (PT) (s) was computed using the Sysmex CS-2500 system (Sysmex Corporation, Kobe, Japan), and the international normalized ratio (INR) was derived using the ISI formula (patient PT/control PT). Regarding hematological parameters, platelet count (10^9^/L) was obtained using the complete blood count analyzer Beckman Coulter LH 780 (Beckman Coulter Ireland Inc., Mervue, Galway, Ireland). Serological tests involved the detection of hepatitis B surface antigen (HBsAg), antibodies to hepatitis B e antigen (anti-HBe), and hepatitis B e antigen (HBeAg) by the ELISA method (Liaison, Diasonin, Italy). Also, HBV DNA was detected by reverse transcription polymerase chain reaction (RT-PCR) (COBAS Ampli Prep/COBAS, TaqMan).

Scores were calculated, APRI: (AST[IU/L]/upper normal limit of AST[IU/L])/platelets [10^3^/mm^3^],


$$\text{FIB}-\text{4}:\;\left(\text{age}\left[\text{years}\right]\;\times \text{AST}\left[\text{IU/L}\right]\right)\text{/}\left(\text{platelets}\left[\text{1}0^{\text{3}}{\text{/mm}}^{\text{3}}\right]\right)\;\times {\text{ALT}}^{\text{1}/\text{2}}\;\left(\left[\text{IU/L}\right]\right).$$

REACH-B scores were calculated using parameters at the initiation of treatment that include factors such as male sex, ALT, status of HBeAg, and level of HBV DNA (Table 1).

**Table 1:** Risk estimates for HCC in chronic hepatitis B score parameters.

		Point
Age	30–34	0
	35–39	1
	40–44	2
	45–49	3
	50–54	4
	55–59	5
	60–65	6
Gender	Male	2
	Female	0
ALT (U/L)	<15	0
	15–44	1
	>45	2
HBV DNA (copies/mL)	<300	0
	300–9,999	0
	10,000–99,999	3
	100,000–999,999	5
	>1,000,000	4
HBeAg	Positive	2
	Negative	0

ALT: alanin aminotransferase; HBV: hepatitis B virus; HBeAg: hepatitis B e antigen.

This study was approved by the institutional review board and the research and ethics committee at Health Sciences University Izmir Tepecik Education and Research Hospital (11.01.2023 and numbered 2022/12-01). The study protocol was conducted in accordance with relevant regulations and guidelines.

The data analysis was conducted using the SPSS 27.0 program with a confidence level of 95%. Descriptive statistics, including mean (mean), standard deviation (SD), minimum (min), maximum (max), and median (M), were reported for the measurements. Kurtosis and skewness values were obtained from quantitative variables. In comparing measurements across determined groups, Mann-Whitney or independent groups t-tests were used for two groups, Kruskal-Wallis or one-way ANOVA for more than two groups, and the chi-square test for assessing the relationship of group variables. The Spearman correlation test was utilized to examine relationships between measurements, while repeated measures ANOVA or Friedman tests were employed for comparing repeated measurements. For pairwise comparison of repeated measurements against the baseline, dependent groups t-tests or Wilcoxon tests were utilized. A p-value of less than 0.05 was considered statistically significant.

## RESULTS

The study included a total of 218 patients. The majority of the patients were male, with a mean age of 49.95±10.91 years. Most of them were anti-HBe positive, and the majority of them had F1 or F2 fibrosis according to liver biopsy. Also, the majority of them (78.4%) were considered treatment-naive. The baseline characteristics of the CHB patients are detailed in Table 2. At the end of the follow-up period, two patients were diagnosed with cirrhosis and one patient with HCC.

**Table 2:** The baseline characteristics of the chronic hepatitis B patients.

		n (%)
Age	<40	39 (17.9)
	40–49	68 (31.2)
	50–59	69 (31.7)
	>59	42 (19.3)
Gender	Male	132 (60.6)
	Female	86 (39.4)
Prior treatment	Naive	171 (78.4)
	Lamivudine	30 (13.7)
	Telbivudine	14 (6.4)
	Adefovir	3 (1.3)
Ishak score (modified Knodell score)	F0	10 (4.5)
	F1	67 (30.7)
	F2	78 (35.7)
	F3	51 (23.3)
	F4	12 (5.5)
Initiated treatment	TDF	76 (34.8)
	ETV	73 (33.4)
	TDF to ETV switch	37 (16.9)
	TDF to TAF switch	14 (6.4)
	ETV to TDF switch	9 (4.1)
	TDF plus ETV	9 (4.1)
HBeAg	HBeAg (+)	61 (27.9)
	Anti-HBe (+)	157 (72)

ETV: entecavir, TDF: tenofovir disoproxil fumarate, HBeAg: hepatitis B e antigen, anti-HBe: antibody to hepatitis B e antigen, F: fibrosis, TAF: tenofovir alafenamide.

The FIB-4 and APRI score measurements exhibited a gradual decrease over the subsequent years of AVT and were found to be statistically significant. In the comparison between time points, the FIB-4 score significantly reduced from the baseline to the first year and from the first year to the tenth year. Although there is no significant decrease from the fifth and tenth years, in the comparison between time points, the APRI score was significantly reduced. The lowest measurements were recorded for each score in the fifth year. While considering tenth-year scores, a statistically significant decrease was observed for APRI scores, compared to the initial measurements, but no significant difference was observed between the tenth-year measurement of FIB-4 scores and the initial measurements (Table 3).

**Table 3:** The evolution of aspartate aminotransferase to platelet ratio index and Fibrosis-4 scores during the treatment period.

		Min–max (M)	Mean±SD	p-value	Initial p-value
FIB-4	Initial	0–8 (1)	1.61±1.2	0.000*	X
	First year	0–4 (1)	1.25±0.58		0.000*
	Fifth year	0–4 (1)	1.25±0.55		0.000*
	Tenth year	0–10 (1)	1.37±0.87		0.074
APRI	Initial	0–9 (0)	0.6±0.92	0.000*	X
	First year	0–1 (0)	0.25±0.12		0.000*
	Fifth year	0–1 (0)	0.22±0.09		0.000*
	Tenth year	0–2 (0)	0.22±0.19		0.000*

*p<0.05 indicates a significant difference, p>0.05 suggests no significant difference. Repeated ANOVA/Friedman test was used for comparison of repeated measurements, and dependent groups t/Wilcoxon test was used for pairwise comparison of repeated measurements compared to the baseline. FIB-4: Fibrosis-4; APRI: aspartate aminotransferase to platelet ratio index; SD: standard deviation. Bold values mean significant difference.

**Figure 1:**
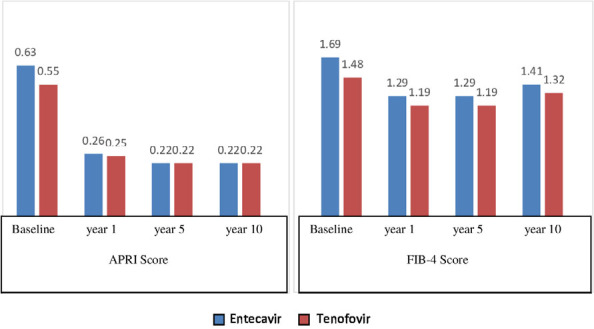
Comparison of alteration in aspartate aminotransferase to platelet ratio index and Fibrosis-4 score measurements over time in treatment groups.

When comparing APRI and FIB-4 scores in the groups receiving ETV and TDF, both groups demonstrated a significant decrease, and no significant difference was observed between the two groups. FIB-4 score measurements exhibited significant changes in the ETV group compared to baseline measurements at all time periods. In contrast, while a significant change in FIB-4 score measurements was observed in the TDF group compared to baseline measurements in the first year, no significant change was noted in the fifth and tenth years. Both FIB-4 and APRI score measurements decreased over time in both groups, reaching the lowest measurements in the fifth year (Figure 1).

Upon analyzing the FIB-4 and APRI scores of the patients at the initiation of treatment, a statistically significant correlation was identified between the FIB-4 and APRI scores, as well as with measurements of histological activity index (HAI), HBV DNA level, AFP level, total bilirubin, and direct bilirubin level (Table 4).

**Table 4:** The relationship between Fibrosis-4 and aspartate aminotransferase to platelet ratio index scores and laboratory features of patients at the initiation of treatments.

		FIB-4	APRI
HAI	r	0.213	0.178
	p	**0.002**	**0.009**
HBV DNA	r	0.212	0.510
	p	**0.002**	**0.000**
Albumin	r	-0.100	-0.122
	p	**0.144**	**0.075**
INR	r	0.042	0.050
	p	**0.555**	**0.480**
PT	r	0.009	-0.033
	p	**0.903**	**0.645**
AFP	r	0.298	0.192
	p	**0.000**	**0.005**
Total bilirubin	r	0.213	0.286
	p	**0.002**	**0.000**
Direct bilirubin	r	0.226	0.295
	p	**0.001**	**0.000**

p<0.05 indicates a significant relationship; p>0.05 suggests no significant relationship. For correlation coefficient ®: 0≤r≤0.25 is very weak, 0.26≤r≤0.49 is weak, 0.50≤r≤0.69 is moderate, 0.70≤r≤0.89 is strong, and 0.90≤r≤1 is very strong. The Spearman correlation test was applied. HAI: histological activity index, INR: international normalized ratio, PT: prothrombin time, AFP: alpha-fetoprotein; FIB-4: Fibrosis-4; APRI: aspartate aminotransferase to platelet ratio index; HBV; hepatitis B virus. Bold values mean significant difference.

Baseline REACH-B scores, indicating the risk of HCC development within 10 years, were calculated for 122 patients who underwent potent AVT for at least 10 years. The calculated scores ranged from a minimum of 0.2% to a maximum of 81.6%, with an average score of 12.33%. Based on these results, it was projected that 15.04 out of the 122 patients would develop HCC within 10 years. However, only one patient developed HCC during this period. The Standardized Incidence Ratio (SIR) was calculated as 0.06, indicating a significant difference between the observed and expected HCC cases, with a much lower incidence than anticipated.

## DISCUSSION

Our research aims to demonstrate the impact of potent AVT on both liver fibrosis and the development of HCC. Existing literature lacks extensive long-term studies exploring the potential risks of HCC and the regression of liver fibrosis. Our study seeks to enrich the literature by providing these findings over an average of 10 years.

A positive correlation was noted between APRI and FIB-4 scores before treatment and various factors such as HAI, HBV DNA level, total bilirubin, direct bilirubin, and AFP levels. However, no significant association was identified with albumin and coagulation parameters. The most robust correlation was found between APRI and HBV DNA levels, which was thought to be compatible with the literature information that necroinflammation and fibrosis are correlated with viral load.

We observed a consistent decrease in both APRI and FIB-4 scores, especially the most rapid decline was observed during the initial year. Notably, no further decrease was observed between the fifth and tenth years of treatment; the APRI score remained unchanged, while there was an increase in the FIB-4 score. The reason for this increase was considered to be associated with one of the parameters of the FIB-4 score system, age of the participants. In a similar study conducted in China, 104 patients who received ETV treatment for 3 years were evaluated, and a significant decrease was observed in APRI (0.45→0.24) and FIB-4 (1.05→0.78) scores at the end of the third year^7^. Tenggara et al. conducted a study involving 41 patients who underwent 1 year of ETV treatment, a notable decrease in the APRI score was observed, dropping from 1.13 to 0.43. Importantly, the regression in the APRI score was found to occur independently of HBeAg status in this study^8^.

In a similar study, Başar et al. included 76 patients using lamivudine, ETV, and TDF regimens, with an average follow-up period of 15.8 months. At the conclusion, a notable reduction was noted in both APRI (0.86→0.72) and FIB-4 (1.53→1.25) scores^9^. Chon et al. conducted a study in South Korea, where a total of 3,277 treatment-naïve patients were monitored for 6 years using ETV and TDF, both APRI (1.46→0.50) and FIB-4 (3.25→2.21) scores exhibited a significant decrease in the noncirrhotic patients. In this study, a statistically significant change was observed in both scores between the baseline and the first year. However, no significant difference was noted between the scores in the first and sixth years^10^. In our study, a noteworthy reduction in both scores was observed between the first and fifth years. Similarly, in the study conducted by Okan et al., which involved 199 patients undergoing ETV, TDF, and lamivudine treatment, both scores were calculated annually. Results indicated a significant decrease during the initial year, with APRI reaching stabilization after the first year and FIB-4 after the second year^11^.

In our study, there was a significant regression of inflammation markers, especially viral load, and a corresponding regression of liver fibrosis. In our investigation, the time-related changes in noninvasive fibrosis scores were compared in distinct treatment cohorts. APRI and FIB-4 score measurements showed significant changes, and no significant difference was observed. Over time, both score measurements reached their lowest points in the fifth year. The APRI score showed a significant reduction in both groups until the fifth year, with no parallel change observed in the tenth year. Similarly, the FIB-4 score experienced a substantial decrease in the first year for both groups, without a comparable change evident in the fifth and tenth years. Specifically in the TDF group, the FIB-4 score in the fifth year did not significantly differ from the baseline value. Notably, the study conducted by Chon et al. demonstrated a significant decrease in APRI scores in both TDF and ETV groups, there is no statistically significant difference between the two groups. In this study, baseline APRI and FIB-4 scores were significantly higher in the ETV group; however, the difference disappeared with treatment^10^. In a study conducted by Yalaki et al., 103 patients were followed under TDF and ETV treatments for 5 years. While the APRI score decreased significantly over time in both groups, unlike our study, FIB-4 did not show a significant change over time in both groups^12^. Another study noted a significant decrease in APRI and FIB-4 within both the ETV and TDF groups after the first year. Specifically, the ETV group exhibited a superior change in FIB-4 score by the end of the initial year^11^. Despite existing studies indicating a greater improvement in APRI and FIB-4 scores during the first year of antiviral treatment in patients receiving ETV compared to TDF, our study did not identify a significant difference between these two groups^11^. This study also included patients using lamivudine^11^. This result, which is contrary to our study, may be due to this situation.

Ideally, assessing the risks of HCC requires a study design incorporating extended clinical follow-up and a control group devoid of treatment. However, considering the fact that the benefits of AVT are undeniable, it creates an ethical challenge to follow patients who are treatment candidates but follow-up without treatment. Consequently, many retrospective studies used some risk scores to predict the onset of HCC. For this purpose, the REACH-B score, developed by Yang et al. in 2011, was used in this study^6^. We observed that with long-term potent AVT, fewer cases experienced HCC than expected before treatment. The median baseline REACH-B score of our participant was 12.33%, and this score was projected to mean that 15.04 out of the 122 patients would develop HCC within 10 years. In a Canadian study involving 322 patients undergoing AVT and with an average follow-up duration of 3.2 years, researchers noted the development of HCC in 11 patients. Significantly, the observed cases were lower than the expected 24 cases based on the initial REACH-B scores, with a SIR of 0.46^13^. In a parallel investigation, a total of 634 patients were monitored, revealing eight cases in the noncirrhotic patient group at the end of the follow-up, compared to the expected 20.11 cases based on initial REACH-B scores (SIR: 0.40). In the cirrhotic patient group, six cases were identified, contrasting with the expected 11.67 cases with initial REACH-B scores^14^. While the REACH-B score was initially developed for noncirrhotic patients and later validated in an external cohort, which included cirrhotic patients, there is limited confirmation of its utility specifically in the cirrhotic patient group. Notably, our study excluded patients with cirrhosis at baseline, eliminating any limitations in this regard. Our study, using the REACH-B scores and SIR, supported previous observations of a lower-than-expected incidence of HCC development over a 10-year period due to AVT. The variations in SIR rates among these studies might be attributed to differences in the number of participants and follow-up durations.

Our study has some limitations: first, it has a retrospective design and was conducted at a single center. Second, there is a need for further research to validate the use of the REACH-B score, initially designed to predict the risk of developing HCC in the absence of treatment, in our population and different age groups, as it was originally developed for the Asian population aged 30–65. The third limiting factor is that the noninvasive fibrosis scores used in our study were initially developed for hepatitis C patients, although both scores have been validated in studies involving CHB patients. The fourth limitation is the lack of histopathological confirmation for noninvasive fibrosis scores calculated at different time intervals. The fifth limiting factor is that the study does not cover different races, and the genotype distribution in the population is uncertain. However, our study makes a significant contribution to the literature by following CHB patients for 10 years and evaluating the fibrosis status with 1.5- and 10-year risk score follow-ups, showing that patients can be followed using a noninvasive method.

This study demonstrates that the impact of potent antiviral treatment on liver fibrosis can be monitored using noninvasive fibrosis scores instead of invasive procedures such as liver biopsy or costly and less accessible methods like transient elastography with a 10-year follow-up period. The other feature is that the use of risk scores eliminates the need for an untreated control group when investigating HCC risk changes. Conducting surveillance for cirrhosis and HCC using noninvasive and cost-effective markers becomes more feasible.
